# Advanced Packaging Techniques—A Mini-Review of 3D Printing Potential

**DOI:** 10.3390/ma17122997

**Published:** 2024-06-19

**Authors:** Anna Witek-Krowiak, Daniel Szopa, Beata Anwajler

**Affiliations:** 1 Department of Advanced Material Technologies, Faculty of Chemistry, Wroclaw University of Science and Technology, 27 Wybrzeze Wyspianskiego Street, 50-370 Wroclaw, Poland; daniel.szopa@pwr.edu.pl; 2 Department of Energy Conversion Engineering, Faculty of Mechanical and Power Engineering, Wroclaw University of Science and Technology, 27 Wybrzeze Wyspianskiego Street, 50-370 Wroclaw, Poland; beata.anwajler@pwr.edu.pl

**Keywords:** additive technology, packaging materials, active packaging, intelligent packaging, insulating packaging material

## Abstract

Packaging and packaging technology constitute a pivotal industry deeply intertwined with our daily lives and prevalent in various settings, including grocery stores, supermarkets, restaurants, and pharmacies. The industry is constantly evolving thanks to technological advances. This article delves into the dynamic landscape of 3D printing in packaging, exploring its profound implications and potential. While this article highlights the advantages of traditional packaging approaches, it also highlights the many benefits of 3D printing technology. It describes how 3D printing enables personalization, rapid prototyping, and low-cost production, streamlining packaging design and manufacturing processes. Offering innovative solutions in design, functionality, and accessibility, the potential of 3D printing in packaging is promising.

## 1. Introduction

The packaging industry has significant environmental and cost impacts throughout the supply chain, including packaging design, material sourcing, and end-of-life processing [1]. The development of packaging began with the need to transport and distribute goods, as the place of production is often different from the place of final use, requiring protection as products move through the supply chain to the point of consumption [2]. Various definitions of the term packaging are given in the literature [2,3,4,5,6]. Most often, it is defined as a finished product having an appropriate structure that is designed to protect the packaged product from the harmful effects of external factors; allows the movement of products during storage, transport, sale, and use; informs about the content through its aesthetics; influences the buyer; and has economic qualities [6]. Packaging and packaging technology are among the most essential branches of the national economy [5]. In the domestic market, products are packaged in various materials: metal, paper, cardboard, plastic, and others [7,8].

In recent years, the packaging industry has grown rapidly [9]. As a result of ever more modern technologies, new generations of packaging are being developed to maintain and even improve the quality of the packaged product. Of all the trends in the packaging industry, 3D printing is at the forefront [10]. While additive manufacturing is not yet the leading method in this area, the cost savings it offers are significant. Three-dimensional printed packaging is an exciting application of the technology [11].

The roots of 3D printing can be traced back to the late 18th century with early innovations in photo-sculptures and topography. In the 1860s, Francois Willeme developed a method to create 3D objects by photographing them from multiple angles and carving the shapes out of wood. Later, in 1892, Blanter introduced a technique for creating topographical maps by layering cut pieces to form 3D structures [12]. Three-dimensional printing, invented by Charles Hull in 1986, is an additive process where materials are layered to form objects based on computer-guided designs [13]. Three-dimensional printers make it possible to prototype packaging at high speed. Designers can iterate through different versions to test shapes and functionality, all in a short period [10]. Three-dimensional printing technology allows virtually any shape to be printed. There are no limitations, whether in terms of geometry or complex shapes [11]. Filaments for 3D printers are designed to mimic different materials, such as wood, glass, or carbon fiber. This allows designers to test different variations and choose the optimal materials. The entire process, from design to production, can be done in house. This reduces product lead times, lowers costs, and minimizes the risk of a new design leaking [14]. Light-curing resin 3D printing technologies are ideal for producing small but accurate models. Automated post-processing systems can be used with 3D printers to produce waterproof, aesthetically pleasing 3D packaging models. The bottom line is that 3D printing in packaging design is not only effective, it is inspiring. It enables the creation of innovative solutions that meet market and customer demands.

Introducing 3D printing technology into innovative food packaging provides several significant advantages that support a transformational impact on the industry. The inextricable link between 3D printing and food packaging innovation lies in the technology’s ability to meet the changing needs of the food industry. Offering personalization, rapid prototyping, sustainability, complex features, cost efficiency, supply chain flexibility, and improved aesthetics, 3D printing is poised to revolutionize the way food is packaged, ultimately improving both the consumer experience and operations [15].

Consumers increasingly demand personalized experiences, and 3D printing can create unique packaging tailored to individual preferences or specific product requirements [16]. Three-dimensional printing enables highly customized packaging designs. This capability is particularly valuable for niche markets, promotions, and personalized branding [17]. Ever-changing consumer preferences and regulatory requirements characterize the fast-paced food industry. Rapid prototyping helps companies remain flexible and responsive to these changes. It accelerates the prototyping process, enabling faster iteration and innovation. Companies can develop, test, and refine packaging designs in a short period. In addition, sustainability is a key issue in food packaging, where traditional methods often result in significant waste. Three-dimensional printing supports the development of sustainable packaging solutions that meet environmental goals and consumer expectations. The additive process can reduce material waste through the precise use of materials and the ability to recycle and reuse them. It also enables the creation of biodegradable and environmentally friendly packaging materials [15]. Three-dimensional printing can produce complex geometries and integrated features that are difficult or impossible to produce using traditional manufacturing methods. For example, enhanced packaging functionality can improve product protection, extend shelf life, and provide a better user experience. Furthermore, smart packaging with embedded sensors, unique shapes, and innovative features can, for example, monitor product freshness and provide real-time information to consumers [17]. Food companies can economically produce small batches of specialized packaging for seasonal products, market testing, or localized offerings without high initial investments. For small or limited production runs, 3D printing can be more cost-effective than traditional manufacturing methods, which often require expensive molds and large production runs. The food industry benefits from a flexible supply chain, particularly in terms of responding quickly to market demands and reducing lead times. One-man manufacturing helps manage supply chain disruptions and maintain product availability. Three-dimensional printing enables on-demand manufacturing, reducing the need for large inventories and minimizing warehousing costs. It can also lead to decentralized production closer to the point of sale or consumption. In a competitive market, visually appealing and distinctive packaging attracts consumers and communicates value [15,16,17]. Three-dimensional printing enables the creation of intricate and aesthetically pleasing designs that enhance the visual appeal of packaging. This can strengthen brand identity and improve product differentiation on store shelves.

In summary, packaging accounts for nearly half of the plastic waste generated worldwide and is the largest market use of plastics [18]. According to [19], only 2% of plastic packaging worldwide is recycled in a closed loop, while the rest is incinerated, landfilled, or ends up in waterways and the environment. To solve this crisis, we need to look at how we design and manage our plastic packaging and how we transition to a closed-loop economy. We need to think differently about plastic at the end of its life: not as waste but as a resource that can be used to make new plastic. This goal should also be a design focus. Once the packaging has served its purpose, the material should enter a closed loop to ensure that it can be reused for the same purpose without degradation. This is where additive technology, known as 3D printing, comes in.

## 2. Three-Dimensional Printed Packages

Of all the trends in the packaging industry, 3D printing is at the forefront [10]. While additive manufacturing is not yet the leading method in this area, the cost savings it offers are significant. Three-dimensional printed packaging is an exciting application of the technology [11]. Some key advantages of using additive technology in this industry are speed of prototyping, unlimited shapes, a wide range of materials, proprietary manufacturing, and small and medium-sized models with simple or complex geometries [20,21].

### 2.1. Overview of 3D Printing Methods for the Food Industry

The most popular technique used in food packaging production is FDM. Due to its availability and low exploitation cost, it is gaining popularity. This 3D printing technique involves extruding a thermoplastic material into a heated nozzle, which, after melting, is applied layer by layer. The main problem and limitation regarding the materials is the necessity of utilizing thermoplastics. It makes producing packaging from biopolymers such as hydrogels highly challenging. Additionally, due to the layered nature of the technique, assembling objects with this method can result in the formation of micropores in the structure [22]. Therefore, assembling packaging that purely increases the freshness of the food is highly problematic, and other alternatives must be sought. Another issue is the strength of the resulting structures since the technique involves layer design through movement in only two planes, X and Y, with no vertical connections between the layers [23]. This type of printing does not allow for the production of advanced structures tailored to any type and shape of food. Due to the low cost of the method and its simplicity, using sealing methods by applying layers that seal the connections of biopolymer composites with thermoplastics has been proposed, which will minimize the drawbacks of this method. While the FDM method is a good foundation to check if producing a type of packaging makes sense, it seems highly unlikely to be used for larger-scale applications [24].

The next technique is semisolid printing, which is ideal for using high-viscosity materials like hydrogels. The material is not preheated and is applied in a similar way to the FDM method but through a peristaltic or syringe model. From the perspective of a broader range of materials, it is a considerably better method for use in food packaging because biodegradable biopolymers can be applied. The main concern with this approach is the need to introduce an additional step of crosslinking the extruded structures through chemical cross-linking or exposure to light or pH [24]. This requires the introduction of an additional production stage, which translates to inflated costs and the necessity to adapt the entire production to a specific type of material. Yet, there is also the problem of utilizing hydrogels, which dehydrate and shrink, losing their mechanical properties, due to temperature. This indicates the demand to introduce additional layers isolating the hydrogel from the external environment, prolonging their properties. Hydrogels are also an excellent environment for microorganism growth, so in the food industry, it is critical to ensure sterile conditions for packaging. In this case, post-production sterilization and then applying an isolating layer, for example, using biopolymers with antibacterial properties, can be proposed. Again, as with FDM, there are no vertical connections between the layers, resulting in low mechanical strength.

Coaxial printing involves the simultaneous extrusion of two different materials, indicating a broader range of applications than FDM and semisolid printing. This technique allows for the production of layered structures during extrusion since the nozzle has two coaxial channels for feeding the material. It is a practical feature considering the difficulties of the semisolid printing method, where it is vital to cross link the extruded hydrogel and isolate it from the environment to improve its properties. This technique can employ high-viscosity materials and biopolymers, indicating its potential in food packaging production. Additionally, the properties to simultaneously extrude two materials allows for minimizing the disadvantages of hydrogels and raising both mechanical and application properties [25]. However, the simultaneous application of materials can lead to production complications related to incompatibility, different shrinkage rates of the materials, and structural defects due to delamination. Besides, the printing method relies on layering material, leading to fundamental structural problems with tightness. Also, controlled, sterile printing conditions are necessary, where materials with antibacterial properties can be applied to the outer layer of the packaging, significantly simplifying the production process compared to the semisolid printing method [26]. The technique allows for packaging with more complex properties and eliminates the drawbacks of its predecessors, indicating the need for intensive research into its application and cost minimization.

Solvent-cast printing concerns feeding a solid polymer material into the machine, which then dissolves the polymer to achieve the desired viscosity. It allows for high control of the material used in packaging production. The process’s idea is to evaporate the solvent during the material application, requiring printing under conditions allowing for quick evaporation, generating significant costs. This causes production problems, as it can lead to deformation, structural gaps, and reduced mechanical strength. The method seems friendly for food packaging because the most commonly used solvent is ethanol, which can be easily evaporated after printing. The material used can be any that is soluble in safe, non-toxic solvents, such as biopolymers [27]. The need to evaporate the solvent might cause the shrinking of hydrogel structures, limiting the method’s application in food packaging. The method itself requires high energy costs associated with solvent evaporation and a slow process due to the need to thoroughly remove solvent residues and their penetration into liquid-absorbing hydrogels, potentially leading to numerous poisonings and food contamination. For these reasons, solvent-cast printing does not seem to be the most optimal solution compared to other available printing methods.

SLS is a 3D printing technique significantly different from the ones mentioned above as it involves sintering layers of the used material with a laser. The method utilizes polymer powder applied to the surface and then sintered with a laser beam, after which the process is repeated until the desired structure is achieved. The method most commonly uses polymers that are not safe for contact with food, limiting its application and often contaminating polymer powders that can penetrate the food. The typically used materials are distinguished by high structural stiffness, and despite the high precision in forming any structure, they are commonly used to produce thick-walled structures, which have no application in food [14,28,29,30]. The process requires a wide range of modifications to adapt the material, removal methods, and surface sealing, indicating the process’s modifiable possibilities, but there are better alternatives for the food packaging market.

SLA is a process utilizing ultraviolet (UV) light to cross link photosensitive polymers. Printing involves immersing the designed platform and coating it with a photopolymer. It is then exposed to UV light, causing it to harden. The next layer is applied by re-immersing and exposing it to light. The method seems friendly for food packaging because it allows the usage of a wide range of photopolymers that are safe for contact with food, and the crosslinking method allows for the creation of advanced structures. However, photo hardening might cause incomplete crosslinking of the polymer, leading to the penetration of toxic compounds into the food. This requires thorough quality control of the resulting products, especially regarding mechanical properties. This is crucial since photo-hardened compounds are typically characterized by low elasticity and susceptibility to impact. SLA allows for creating detailed structures but requires careful planning during the design stage due to the shrinkage of polymers during crosslinking, and after the structure is made, it must be re-exposed to UV light to crosslink any residues to avoid food contamination [14,28,29,30]. The SLA method shows significant application potential but requires material engineering research and adaptation of polymers for contact with food and coatings to enhance mechanical properties.

The last discussed technique is SLM, which follows the same principle as SLS, involving the usage of a laser to completely melt the used powder, which was not the case in SLS. The method typically employs metals, meaning it can be used for packaging that does not have direct contact with food due to contamination. Yet, there is a possibility of using bio-compatible metals for packaging. The method allows for the best precision in assembling structures and mechanical strength. Therefore, the resulting structures are rigid, which is not a good feature for food packaging [26]. Additionally, it is a process requiring high operational costs, making it not worth considering for food packaging production. It is worth considering combining this method with cheaper solutions to reduce costs and expand the application range.

In summary, the best method in terms of application potential and structural modification possibilities is coaxial printing, which represents an evolution of the most commonly used 3D printing method, FDM. Expanding research on the safety of the biopolymers used and prevention of contact with the environment to limit losses in mechanical properties and contamination by microorganisms is proposed.

**Figure 1:**
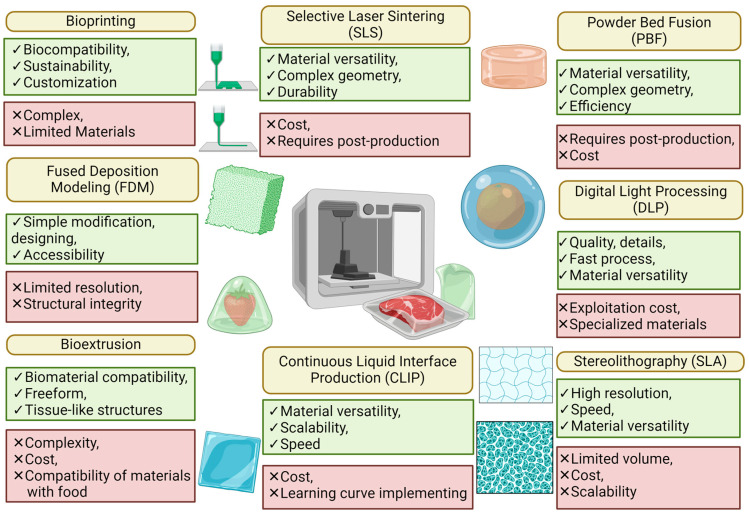
A compilation of 3D printing techniques with promising applications in the manufacturing of food packaging. Created with Biorender.com.

Three-dimensional printing has found use in applications for designing, manufacturing, and evaluating the properties of various structures that allow for its application as food packaging. Three main scenarios dominate this group: active packaging, intelligent packaging, and insulating properties (Table 1). Research on the scope of 3D printing is relatively new, and the use of all methods to create food packaging with a wide range of applications has not yet been explored. FDM techniques are most often used, but there are also many other techniques of note presented in Figure 1. This highlights the necessity for thorough research to assess the suitability of each technique for packaging creation given the specificity of processes and the requirements of particular groups, packaging types, and preparation methods. Notably, techniques such as SLA, DLP (renowned for high precision and complex structure fabrication), CLIP (known for its speed and continuous process), and PBF (recognized for producing highly durable packaging) stand out. However, limitations prevail due to a lack of proper materials, emphasizing the importance of ongoing research into optimization and the exploration of new food-safe materials. These research types are key for advancing the application of 3D printing in packaging production.

### 2.2. Active Food Packaging

Active food packaging is a solution that allows for the secretion or capture of chemicals to ensure that the freshness of the food product is maintained, thus extending its shelf life. Compounds that can prevent spoilage are primarily various ingredients with antimicrobial and antioxidant effects as well as compounds capable of capturing gases or aromas released by food [15]. Particular attention is paid to materials of biological origin, especially from renewable raw materials (by-products or waste from various industries).

Polymeric active films are mainly produced by solvent casting. The researchers decided to overcome the problems associated with the traditional casting of edible films (long drying time, lack of thickness control, difficulty of scale up) by taking advantage of 3D printing capabilities using semi-solid extrusion [22]. The study examined the influence of different nozzles (23 G, 25 G, 30 G) and solution feeding pressure from 0.020 to 0.062 MPa. The remaining parameters were constant, i.e., nozzle height was 0.2 mm and feeding speed was 5 mm/s. Soy protein isolate-based printing is a promising direction in developing packaging films as an alternative to plastic packaging. The optimization of response surface method print manufacturing allows for the selection of the most favorable conditions for the production of edible films, including protein concentration, plasticizer, and drying time, while the system response is the film thickness, tensile strength, and puncture resistance. In this case, an extrusion-based 3D printing model was used. A 4.0 mm nozzle was utilized with a 2200 mm/min print speed, and the ingredient flow speed was 0.5 in the optimization cycle [39].

Active components can be introduced into the protein solution, giving the film additional functions: protective and biologically active solutions. Plant polyphenols are a group of compounds that exhibit broad biological activity. They can be derived from available raw materials using extraction methods. Due to the abundant presence of hydroxyl groups, polyphenols can interact with polymer matrices, strengthening their structure. On the other hand, complexes of polyphenols with proteins (e.g., soy protein) can favorably improve the stability of polyphenols [40]. Researchers used polyphenols from green tea and grape seeds to produce edible soy protein-based films by 3D printing. This work analyzed the effects of printing parameters such as printing nozzle size, printing pressure, and polyphenol concentration on the resulting films’ physicochemical properties [22]. The large proanthocyanidins present in the grape seed extract significantly increased the viscosity of the protein solution, which involved strong interaction between these components and caused changes in print quality but positively affected mechanical properties.

Other protein biopolymers like gelatin are also excellent for producing edible active films. *Garcinia atroviridis*, commonly used in Asian cuisine, has antimicrobial and anti-inflammatory properties, and its extract can extend shelf life. Printed edible films with added extract (in the range of 1–4% by weight) were generated using an extrusion printer at 30 °C. Films with a higher extract content had higher water solubility and reduced mechanical strength, indicating interactions between the polyphenols in the extract and the functional groups of gelatins [23] Printed films based on gelatin and cornstarch with the addition of hawthorn berry extract (up to 10% m/v) were evaluated for mechanical and antimicrobial properties. The most favorable effect against *P. aeruginosa* was obtained for films with extract concentrations above 6%. Such an extract content also ensured that proper tensile parameters were obtained [24]. In the formation of active films, gelatin can interact with other polymers, such as polysaccharides. A mixture of gelatin and ZnO nanoparticles was used together with a solution containing an antimicrobial component (clove oil) as a semi-solid extrudate for 3D printing. The prepared mass was mixed with a sodium alginate solution and extruded into a crosslinking agent solution containing Ca^2+^ ions [31].

Composites of chitosan, halloysite nanotubes, and tea polyphenols were used to produce the film by casting, and then the mixture was also subjected to film printing [25]. Based on this formula, the same authors undertook the printing of an active storage package for blueberries, which succeeded in prolonging their freshness (less weight loss and inhibition of rot). This effect was probably due to the presence of chitosan with antimicrobial properties and tea polyphenols also showing biological activity [32].

Zhou et al. (2021) prepared a dual-function active packaging, which was supposed to have cushioning properties (preventing mechanical damage to food during transportation) and antimicrobial properties to delay post-harvest degradation of fruits and vegetables [26]. They proposed coaxial printing of two inks, an inner one consisting of chitosan and silver nanoparticles and an outer (shell) one that contained carboxymethylcellulose, glycerin, and acrylamide derivatives. A coaxial needle with a diameter of 17/22 G was used, and the solution was administered at a speed of 4 mm/s, after which the solution was applied and cross-linked with UV light for 7 min. Coaxial 3D printing produced a three-dimensional structure, which was then lyophilized. The prints had good elasticity and cushioning properties and were biodegradable. The presence of silver nanoparticles promoted antibacterial properties against *E. coli* and *S. aureus*, which is associated with the controlled release of nanoparticles from the material. Other researchers printed antibacterial Ecoflex films prepared using silicon and carbon nanoparticles (extracted from rice husk) and silver dispersed in a polymer [33]. Due to the presence of AgNP, the films showed good bacteriostatic properties, and no release of nanoparticles from the films was demonstrated in tests over a period of 4 weeks, both in the aqueous environment and in food. Other researchers produced and tested prints based on PLA and metallic microparticle powder [34]. Prepared fibers containing copper, aluminum, bronze, and stainless steel were subjected to printing on small flat sheets. All prints had antimicrobial activity, but the sheet containing 90% Cu showed the best antimicrobial activity against the tested strains after just 20 min (99.99% inhibition). The same researchers went on to produce composites with antimicrobial properties using alternately printed PLA layers containing copper, steel, or copper and aluminum. In both works, the FFF 3D printing technique was used, where a structure was printed with a thickness of 1 mm, a width of 40 mm, and a length of 40 mm. The printing process included maintaining the nozzle temperature at 210 °C, where constant fan air was required to set the compound. The compound was applied at a speed of 45 mm/s, where walls were applied with a height of 0.2 mm until they reached 1 mm [35]. The composites produced showed even better activity against gram-positive and gram-negative bacteria.

Active food packaging is an innovative solution that uses a variety of chemicals to ensure longer shelf life and freshness of food products. Ongoing research focuses on using bio-based materials, such as soy protein, gelatin, or chitosan, which have antimicrobial and antioxidant properties. Thanks to 3D printing technologies and the optimization of production processes, it is possible to create active films or other forms (cubes, sheets) with additional biologically active functions. The use of plant polyphenols, silver nanoparticles, or metallic microparticles makes it possible to obtain packaging with antimicrobial properties, which contributes to the further development of the food packaging industry while providing greater product protection and a longer shelf life.

### 2.3. Intelligent Food Packaging

Intelligent packaging includes three main groups of solutions, including freshness indicators, sensors, and information carriers [41]. Indicators most often provide information about changes in the food associated with a loss of freshness. They are prepared in the form of colored markers that undergo a color change under the influence of a stimulus, which allows for visual assessment of the appearance of a food safety hazard. The indicators can react to the presence of metabolites resulting from the growth of microorganisms (e.g., carbon dioxide, volatile nitrogen compounds, organic acids), changes in the pH of the spoiled product, or temperature changes during storage. Through the use of such solutions, food waste losses can be significantly reduced. Intelligent solutions have a communication function, enabling a facilitated flow of information from producers to customers, along with information about the quality of the product that is on sale [42]. This avoids the stress of buying poorly stored products that may spoil before the expiration date or throwing away a product that is still fresh and going to waste after the expiration date.

Four-dimensional printing increases the range of application possibilities of 3D printing by adding a function that changes over time related to the response to an external stimulus. Such a function could be, for example, a color change, which is characteristic of applications in active packaging. Labels indicating the freshness of a product should be made of food-safe materials and, preferably, should contain compounds of natural origin. An excellent indicator of freshness are various plant dyes, such as anthocyanins, which are sensitive to changes in pH and can inform about changes inside the food package. Anthocyanins are ideal for monitoring the freshness of meat products due to their sensitivity to the presence of alkaline volatile amines, which are secreted from spoiled meat. Anthocyanins can be extracted from many raw plant materials, but despite their colorimetric properties, they do not have good food preservative properties [36]. Therefore, it is necessary to use additional compounds to achieve the expected shelf-life extension effect. One publication used the FDM 3D printing method using a cylindrical shape injector with a diameter of 30 mm. Water in oil emulsions (volume ratio of 2:8) with the addition of 1% agar was used, where the emulsion was administered through an extruder with a diameter of 0.8 mm at a speed of 15 mm/s. Li et al. (2022) prepared a printed dual-function film based on chitosan-containing lemongrass essential oil with antioxidant and antimicrobial properties and anthocyanins to monitor freshness via color change. The print was reinforced with a second layer of chitosan film. The printing pressure was selected at 40 kPa, ensuring the best film structure. The films changed color from purple to gray-blue at a pH above 6, allowing for visual detection of pork spoilage using a color measurement smartphone application. The FDM printing method was also used with the following printing parameters: 0.6 mm height, 0.3 mm width, and four layers. The filling density was set at 100%, and the entire process was carried out at a temperature of 25 °C [37].

Zhou et al. (2021) developed a bifunctional label to inform about freshness and, at the same time, prevent food spoilage. The informational part, a colorimetric indicator of pH changes, was equipped with anthocyanins; the food preservative part contained 1-methylcyclopropene, an ethylene receptor inhibitor that inhibits fruit ripening and spoilage. The whole thing was produced by coaxial 3D printing in a polysaccharide hydrogel (sodium alginate, chitosan, carrageenan) containing cellulose nanofibers, in a core-shell form, in which the preservative component was inside, and the informative component was located outside [26]. Different printing parameters were used depending on the packaging application. In the case of 3D-printed cushioning packaging, aerogels were prepared by uniaxial printing technology, where the solution were applied by a 16 G needle (1.26 mm) before being subjected to UV light for 7 min and freeze dried. For packing with antibacterial properties, a coaxial printing method was utilized. The solution was extruded by a coaxial needle (17 G/22 G) at a speed of 4 mm/s, and then the structure was subjected to UV light and freeze dried. The materials were freeze dried and submitted for storage tests with lychee fruit. A label containing a preservative ingredient and anthocyanins extended the fruit’s freshness for up to 6 days, reporting changes in freshness. Another proposed solution to reduce anthocyanins’ leaching from the sensor surface is a two-part design involving a hydrophilic-oleophilic bigel [36]. Potato anthocyanin-containing agar was immobilized in the inner zone and surrounded by a hydrophobic layer containing vegetable oil, beeswax, and glyceride monooleate. The double W/O system was printed on PVDF film and tested successfully for trimethylamine sensitivity under model and real conditions during the storage of beef and salmon.

Moisture sensors can be printed from a blend of polylactic acid and polyethylene oxide (PLA/PEO). The addition of PEO increases hydrophilicity and improves elastic properties, which also affects the detection of high moisture content (increased transparency). The addition of cobalt salts allowed for colorimetric determination of changes in moisture content in the tested environment. In this experiment, it was applied by inkjet printing to the printed PLA/PEO polymer skeleton. The printed skeleton had a porous and highly hydrophilic surface, which ensured good ink distribution and, thus, the observation of a color change (from blue to light pink in the presence of moisture in the range above 60% RH). In this case, solvent-cast printing was performed; with this method, it is possible to precisely adjust the viscosity of the applied compound, which was set at 1.0 dL/g. During application, a feeding speed of 25–30 mm/s was used at a pressure of 3–4.5 bar [27].

Three-dimensional manufactured smart packaging is a tailor-made solution for the food industry. Thanks to the mobilization of components that inform about environmental changes, consumers can be easily assured of the current state of food. Incorporating additional freshness-prolonging materials (e.g., components with antioxidant and antimicrobial properties) makes producing smart packaging with extended functional properties possible.

### 2.4. Thermal Insulations

**Figure 2:**
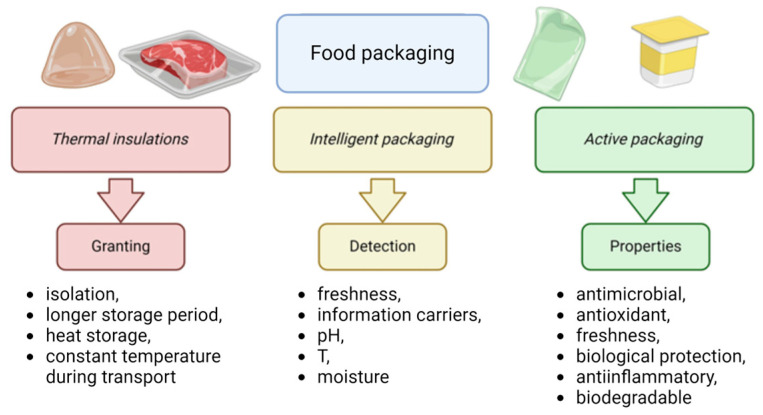
Effects of different types of packaging. Created with Biorender.com.

Three-dimensional printing allows for the formation of three-dimensional structures with any spatial arrangement, so packaging with excellent insulating properties can be designed. For example, such packaging can be used to store and transport frozen food. Porous printed structures filled with air or other gases can be used as thermal insulation packaging. The core can be formed into closed or open foam or periodic structures. Air baffles are spaces filled with air or inert gases, thanks to which the insulation’s expected heat transfer parameters can be achieved. Researcher Anwajler [14,28,29,30,43,44,45] has conducted extensive research on printed insulation materials, studying variations in polymer composites in FDM, SLS, SLA, and DLP technologies. Her analysis focused on the effects of the infill structure, the materials used, and the layering of the composites on their insulating performance. Her research has shown that the printed composites can be effectively used as insulating materials, achieving thermal conductivity coefficients as low as 0.023 W/(m·K). Other materials can serve as energy accumulators, such as phase change materials (PCMs), in which heat can be stored during the phase transformation of such materials and released under different conditions. Such materials can be various organic compounds, including eutectic mixtures, waxes, or paraffins. Their thermal conductivity can be problematic, which is why composite PCMs with metal foam are used, which can reduce the time of heat storage, even by more than 40%, which has been proven using aluminum foam with a paraffin. The SLS technique was used, which, thanks to the principle of combining packaging material with a laser beam, makes it ideal for creating temperature sensors [38]. The ability to select a variety of materials that build up the porous structure offers a wide range of possibilities, including improved thermal performance when using metals with high thermal conductivity, such as copper [46] (Figure 2).

### 2.5. Three-Dimensional Printing from Waste Biomass

Food waste can be a source of raw materials for 3D printing, providing a complete valorization. Stable prints were obtained from banana peel powder with guar gum by extrusion adjusting printing pressure, printing speed, and nozzle height [47]. The same researchers also produced printed packaging from ground rice husk with guar gum [48] and prints from banana peel waste with sugarcane bagasse (also with guar gum) [49]. Added to blends, guar gum improves fluidity and thus print quality. Chitin, a polymer extracted from waste (invertebrate shells), and its derivative, chitosan, can reduce stiffness and increase the plasticity of other materials used in 3D printing, including PLA [50]. Pineapple leaf fiber can also be used as an additive in 3D PLA printing in powdered form as well as after alkali treatment, and the resulting composites strengthen the structure of the prints [51]. Walnut shells, being cellulose- and lignin-rich fillers, can also be added to PLA. However, it is necessary to functionalize the shell particles beforehand by chemical modification with silane [52]. Also, starch, a residue material in the agricultural sector, can be a raw material for food packaging by 3D printing. The use of chachafruto starch to produce biodegradable films using this technique suggests that these products can be used as packaging for products with medium moisture content [53].

The 3D printing process also allows expansion into other agricultural wastes, enabling the development of unusual and biodegradable food packaging. As a result, the method supports the replacement of plastics with sustainable agricultural materials. Given the numerous opportunities for waste processing, 3D printing is becoming an important tool in promoting sustainability in this sector.

## 3. Conclusions and Future Perspectives

Personalizing packaging designs with 3D printing opens up new possibilities for customizing packaging, enabling the creation of unique and recognizable products for specific brands. This process, based on producing forms of any geometry through one-step additive processes, reduces material consumption, requires minimal control, and generates minimal waste. The decreasing cost of 3D printers is a key factor facilitating the development of this technology in the packaging industry, aligning with modern industrial concepts like Industry 4.0. Active packaging is an innovative solution in the food industry, but its implementation poses technological challenges and requires consumer acceptance. One major challenge is adapting 3D printing technology to produce active packaging, which needs special properties such as releasing active substances or responding to environmental changes. Constant exploration and development of new methods and materials are necessary to ensure active packaging effectively extends the shelf life of food products. The prospect of 5D and 6D printing technologies presents even greater opportunities, enabling the production of complex-shaped food packaging and using smart materials, potentially revolutionizing packaging design. Intelligent packaging, which monitors product freshness and reduces food waste, contributes to greater efficiency in the food production industry, though safety standards and consumer acceptance remain crucial issues. The use of waste materials for 3D printing packaging is a sustainable approach that addresses environmental concerns and resource efficiency. However, limitations such as the hydrophilic nature of biopolymer films and their mechanical properties must be overcome. The rapid development of 3D printing in food packaging promises innovative solutions to protect food products effectively, convey relevant product information, and integrate protection and communication functions, significantly raising safety standards and consumer awareness. Porous print structures for heat insulation and phase change materials in printed metal foams for heat storage illustrate the potential of 3D printing in this sector. Thus, 3D printing contributes to logistical efficiency, food safety, and sustainability, positioning itself at the forefront of the food industry’s future evolution.

## Figures and Tables

**Table 1:** Various materials used for 3D printing with packaging applications.

Application	3D Printing Method	Materials	Shape	Function	References
Active packaging	FDM	soy protein isolate, grape seed extract, green tea extract	film	edible active packaging	[22]
	FDM	gelatin, *Garcinia atroviris* extract	film	edible active packaging	[23]
	FDM	corn starch, gelatin, hawthorn berry extract	film	edible active packaging materials	[24]
	semisolid FDM	gelatin, sodium alginate, ZnO, clove essential oil	film	active packaging	[31]
	FDM	chitosan, halloysite nanotubes, tea polyphenols	cuboid, 3D container	active packaging	[32]
	FDM	chitosan, halloysite nanotubes, tea polyphenols	film	active packaging	[25]
	coaxial FDM	carboxymethylcellulose, glycerin and acrylamide derivatives, chitosan, AgNP	film	active packaging	[26]
	FDM	Si-C-Ag-NP, polymer	film	active packaging	[33]
	FFF	PLA, Cu, Al, stainless steel, bronze	flat sheet	active packaging	[34]
	FFM	Cu-PLA-SS and Cu-PLA-Al	flat sheet	active packaging	[35]
Intelligent packaging	coaxial hydrogel FDM	sodium alginate, chitosan, carrageenan, nanocellulose,	cuboid	freshness indicator with properties preventing food spoilage—anthocyanin–visual changes, 1-methylcyclopropene-preservation	[26]
	FDM	agar, beeswax, oil, glyceride monooleate	film layer	freshness indicator—volatile amines sensor (anthocyanins)	[36]
	solvent-cast 3D printing	PLA/PEO	mesh	colorimetric humidity sensor	[27]
	hydrogel FDM	chitosan, lemongrass oil, starch	film	freshness indicator with properties preventing food spoilage—anthocyanin–visual changes, lemongrass oil-preservation	[37]
Insulations	SLS, SLA, FDM	polyamide PA-12, PLA, UV Resins	cuboid	thermal insulations	[14,28,29,30]
	SLM	aluminum foam-paraffin	cuboid	heat storage applications	[38]
